# Supplementary material for: Mechanism of co-transcriptional cap snatching by influenza polymerase
Source: Nature. 2026 Mar 4;652(8112):1281–8. doi: 10.1038/s41586-026-10189-0 (PMC13128444; doi:10.1038/s41586-026-10189-0)

nature portfolio

https://doi.org/10.1038/s41586-026-10189-0

Supplementary information

Mechanism of co-transcriptional cap snatching by influenza polymerase

In the format provided by the authors and unedited

Supplemental File 1 – Uncropped Gel Images

Figure 1b

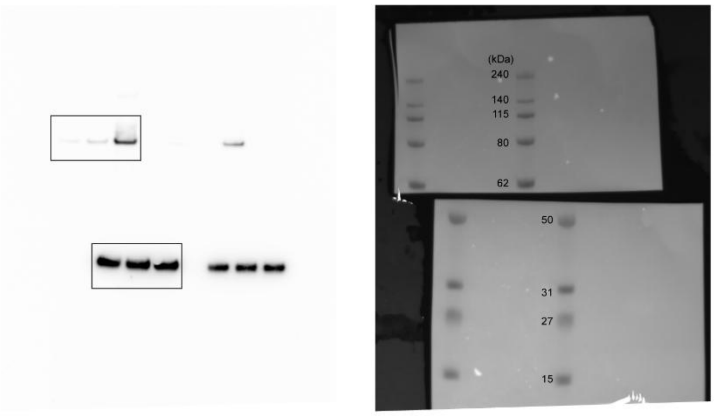

ED Figure 1a

Pol II

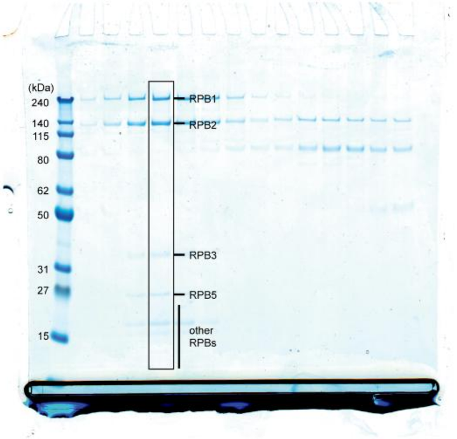

H7N9 FluPol

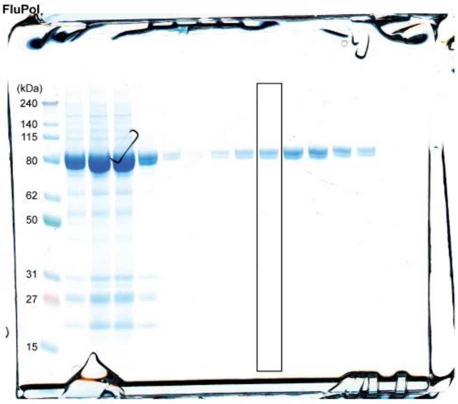

H7N9 FluPol PA E119D

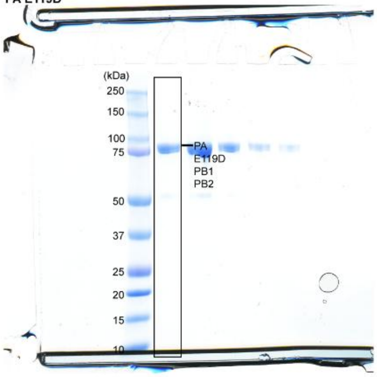

DSIF

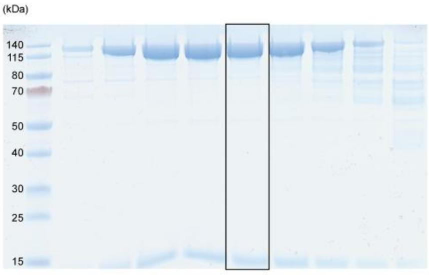

CAK

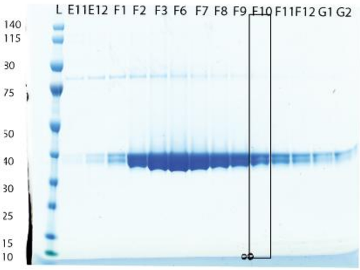

ED Figure 1b

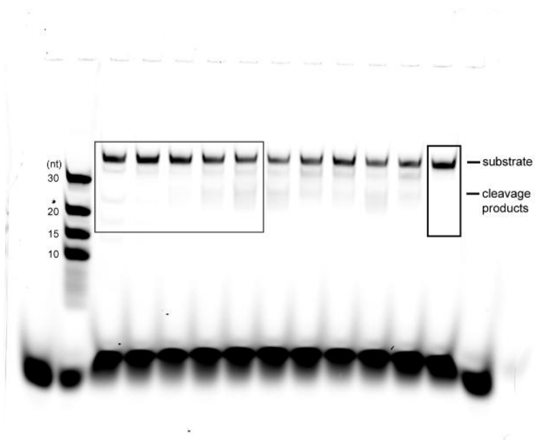

ED Figure 1d

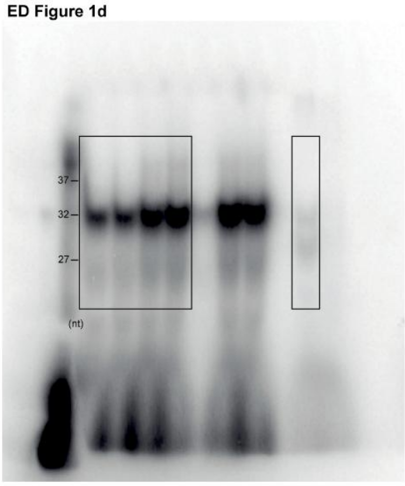

ED Figure 1e

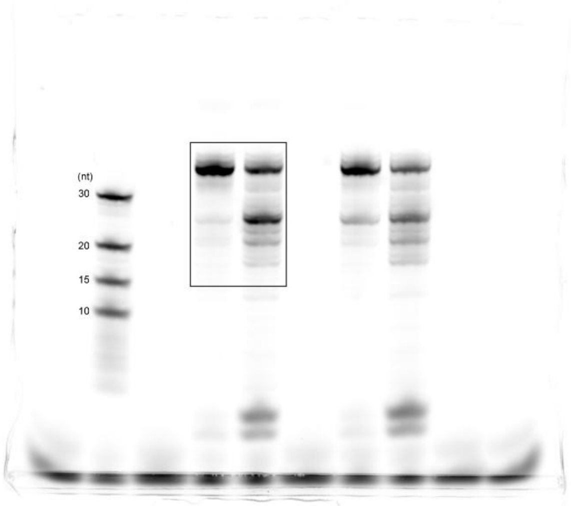

ED Figure 3k

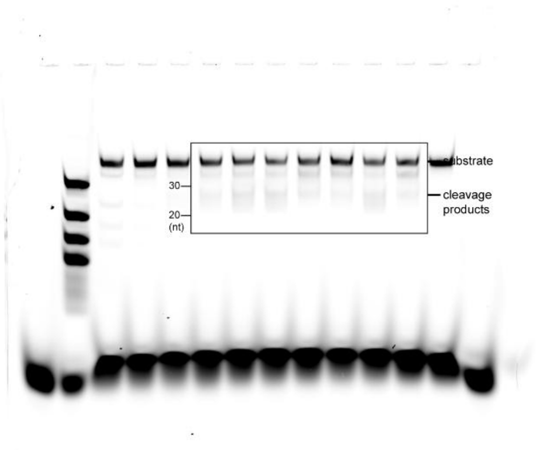

ED Figure 5a

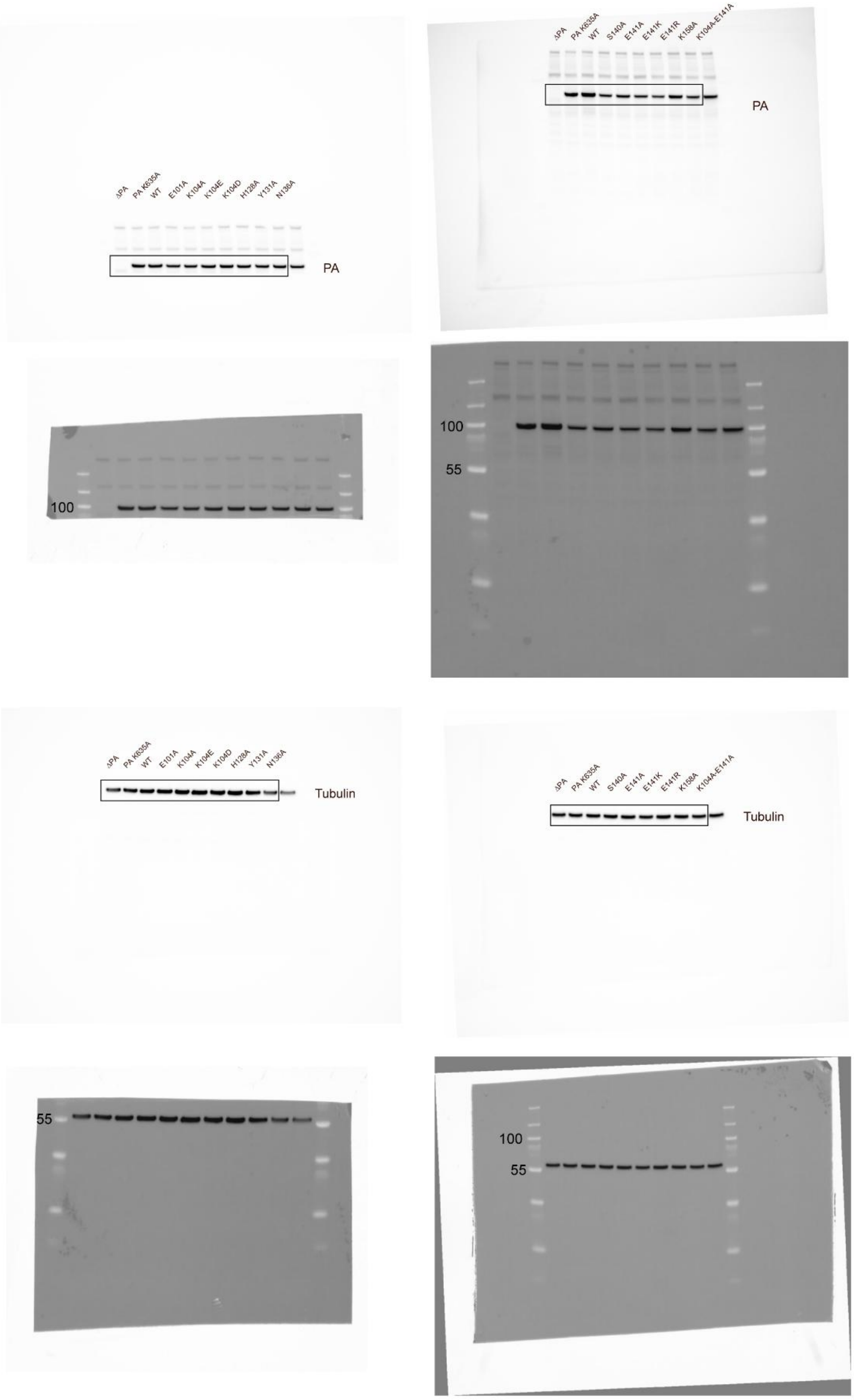
ΔPA
PA K635A
WT
E101A
K104A
K104E
K104D
H128A
Y131A
N136A
PA
ΔPA
PA K635A
WT
S140A
E141A
E141K
E141R
K158A
K104A-E141A
PA
100
100
55
Tubulin
Tubulin
55
100
55

ED Figure 5b

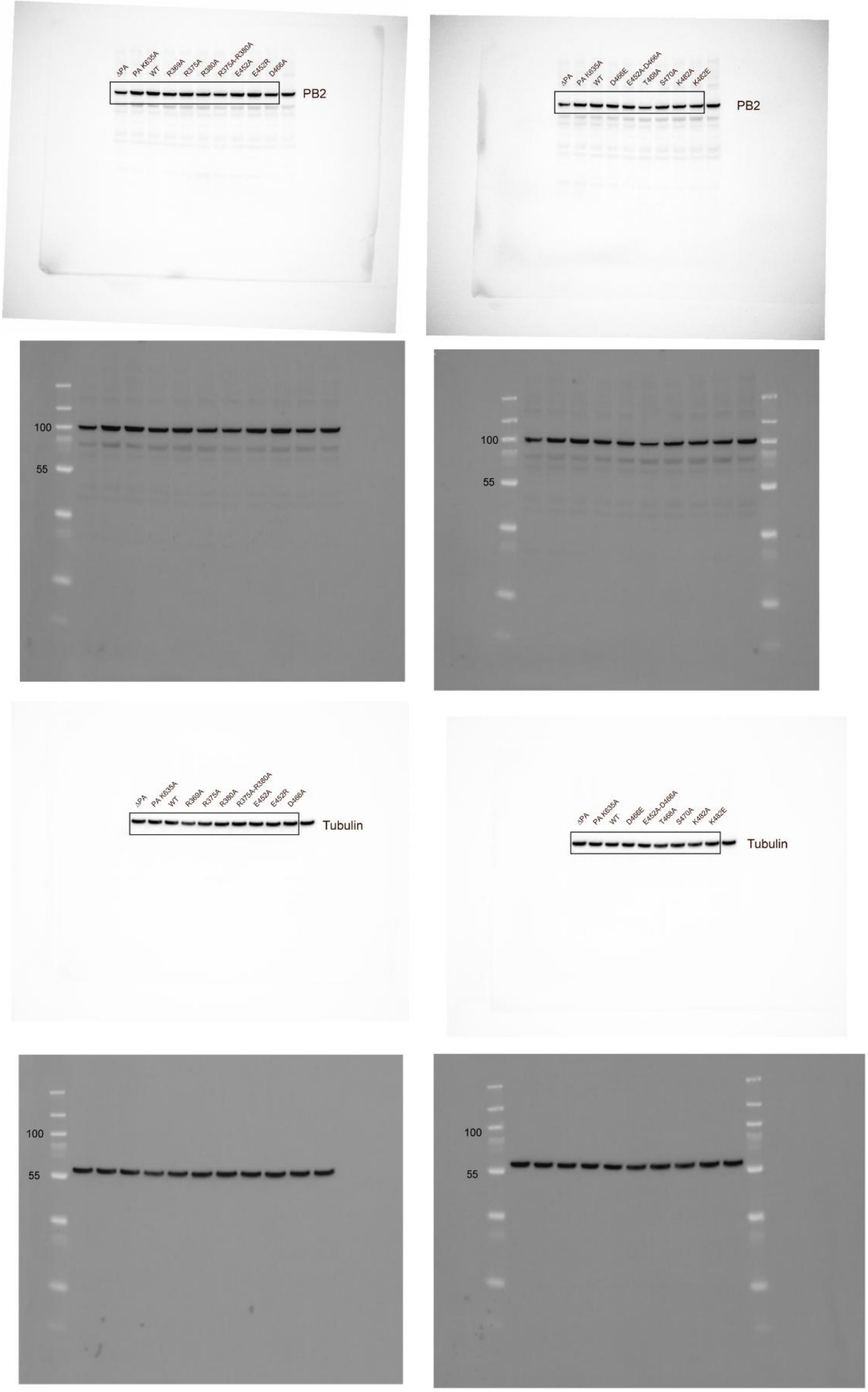
ΔPA
PA K635A
WT
R369A
R375A
R380A
R375A-R380A
E452A
E452R
D466A
PB2
ΔPA
PA K635A
WT
D466E
E452A-D466A
T468A
S470A
K482A
K482E
PB2
100
55
100
55
ΔPA
PA K635A
WT
R369A
R375A
R380A
R375A-R380A
E452A
E452R
D466A
Tubulin
ΔPA
PA K635A
WT
D466E
E452A-D466A
T468A
S470A
K482A
K482E
Tubulin
100
55
100
55

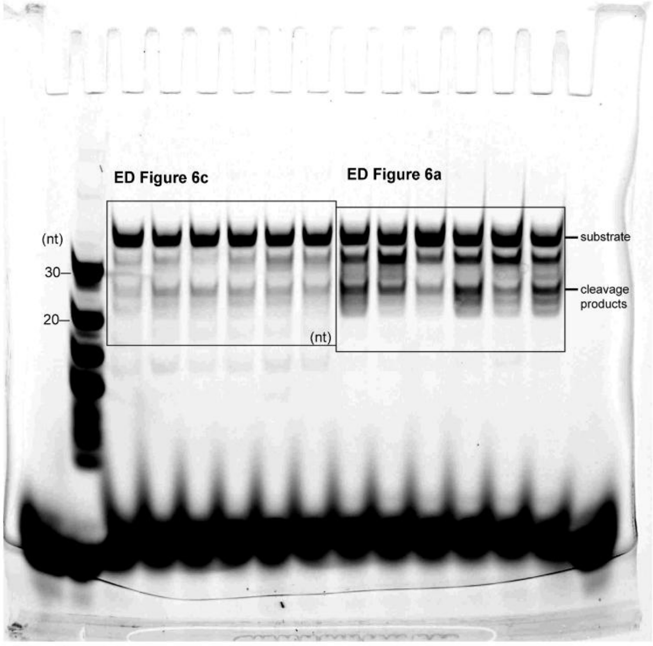

ED Figure 6d

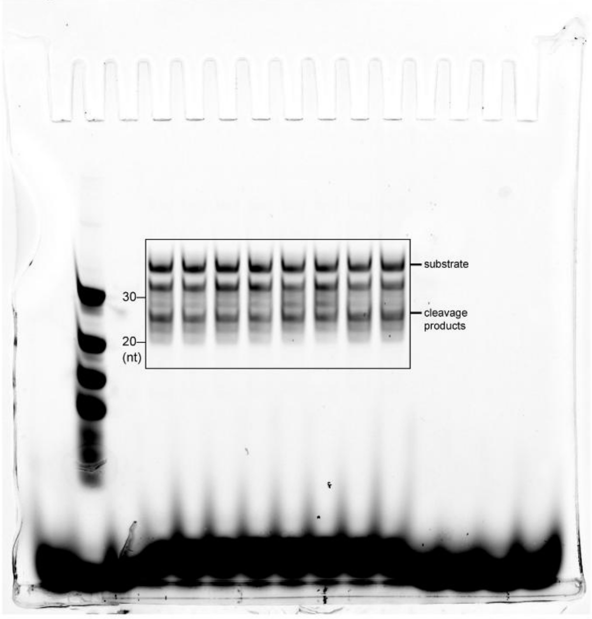

ED Figure 6f

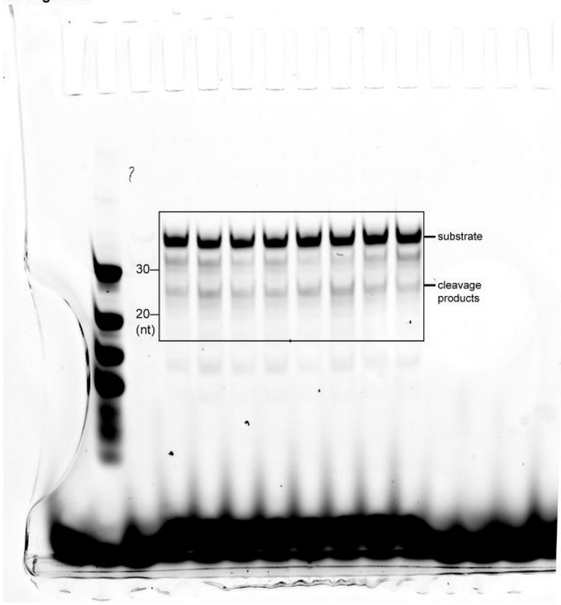

Supplement: Supplementary file 1 — The uncropped gel images. [file 41586_2026_10189_MOESM1_ESM.pdf]
