# Supplementary material for: Mechanism of co-transcriptional cap snatching by influenza polymerase
Source: Nature. 2026 Mar 4;652(8112):1281–8. doi: 10.1038/s41586-026-10189-0 (PMC13128444; doi:10.1038/s41586-026-10189-0)
Supplement: Supplementary file 2 — Reporting Summary [file 41586_2026_10189_MOESM2_ESM.pdf]

nature portfolio

Corresponding author(s): Patrick Cramer, Christian Dienemann, Nadia Naffakh, Stephen Cusack

Last updated by author(s): 10/10/2025

Reporting Summary

Nature Portfolio wishes to improve the reproducibility of the work that we publish. This form provides structure for consistency and transparency in reporting. For further information on Nature Portfolio policies, see our Editorial Policies and the Editorial Policy Checklist.

Statistics

For all statistical analyses, confirm that the following items are present in the figure legend, table legend, main text, or Methods section.

n/a	Confirmed	
☐	☒	The exact sample size (*n*) for each experimental group/condition, given as a discrete number and unit of measurement
☐	☒	A statement on whether measurements were taken from distinct samples or whether the same sample was measured repeatedly
☐	☒	The statistical test(s) used AND whether they are one- or two-sided<br>*Only common tests should be described solely by name; describe more complex techniques in the Methods section.*
☒	☐	A description of all covariates tested
☐	☒	A description of any assumptions or corrections, such as tests of normality and adjustment for multiple comparisons
☐	☒	A full description of the statistical parameters including central tendency (e.g. means) or other basic estimates (e.g. regression coefficient) AND variation (e.g. standard deviation) or associated estimates of uncertainty (e.g. confidence intervals)
☐	☒	For null hypothesis testing, the test statistic (e.g. *F*, *t*, *r*) with confidence intervals, effect sizes, degrees of freedom and *P* value noted<br>*Give P values as exact values whenever suitable.*
☒	☐	For Bayesian analysis, information on the choice of priors and Markov chain Monte Carlo settings
☒	☐	For hierarchical and complex designs, identification of the appropriate level for tests and full reporting of outcomes
☒	☐	Estimates of effect sizes (e.g. Cohen's *d*, Pearson's *r*), indicating how they were calculated

Our web collection on statistics for biologists contains articles on many of the points above.

Software and code

Policy information about availability of computer code

Data collection	Amersham Typhoon Scanner 3.0, Serial EM 4.0, Roche LightCycler® 480 system v1.5.0.39
Data analysis	cryoSPARC 4.3.1, RELION 3.1, Warp 1.0.9, PHENIX 1.19.2, UCSF Chimera X-1.6, ISOLDE 1.6, Coot 0.9.6, ImageJ 2.9.0, Prism 9.4.1, Snapgene 7.0.1

For manuscripts utilizing custom algorithms or software that are central to the research but not yet described in published literature, software must be made available to editors and reviewers. We strongly encourage code deposition in a community repository (e.g. GitHub). See the Nature Portfolio guidelines for submitting code & software for further information.

Data

Policy information about availability of data

All manuscripts must include a data availability statement. This statement should provide the following information, where applicable:

- Accession codes, unique identifiers, or web links for publicly available datasets
- A description of any restrictions on data availability
- For clinical datasets or third party data, please ensure that the statement adheres to our policy

The electron density reconstructions and final models were deposited with the EM Data Bank (accession codes 50892, and 50927) and the PDB (accession codes PDB 9FYX (pre-cleavage), and 9G0A (post-cleavage)).

Research involving human participants, their data, or biological material

Policy information about studies with human participants or human data. See also policy information about sex, gender (identity/presentation), and sexual orientation and race, ethnicity and racism.

Reporting on sex and gender	not applicable
Reporting on race, ethnicity, or other socially relevant groupings	not applicable
Population characteristics	not applicable
Recruitment	not applicable
Ethics oversight	not applicable

Note that full information on the approval of the study protocol must also be provided in the manuscript.

Field-specific reporting

Please select the one below that is the best fit for your research. If you are not sure, read the appropriate sections before making your selection.

☒ Life sciences ☐ Behavioural & social sciences ☐ Ecological, evolutionary & environmental sciences

For a reference copy of the document with all sections, see nature.com/documents/nr-reporting-summary-flat.pdf

Life sciences study design

All studies must disclose on these points even when the disclosure is negative.

Sample size	For endonuclease assays we choose the sample sizes based on the samples size of the simplified assay in the first draft. Since we observe a reproducible and statistical significant difference for the CTD phosphorylation (positive control) we assume the sample size is sufficient.<br>For cryo-EM experiments, several million initially picked particles were collected, in line with previous published cryo-EM experiments on Pol II. Since single particle cryo-EM experiments requires averaging over thousands of particles, the sample size was sufficient.<br>Sample sizes for cell-based assays were estimated on the basis of previous studies using similar methods and analyses that are widely published, for example see Krischuns et al., 2024.
Data exclusions	We excluded broken particles and poorly aligned particle classes. This is routine practice in cryo-EM to exclude such particle because they introduce noise in the reconstruction. One out of 5 qPCR experiments was taken out, because it was performed with a batch of dNTPs that was later found to be flawed. One of the endonuclease assays on FluPol mutants was removed, as an RNAse contamination was detected in one of the samples in a time point zero control sample. One of the initial 7 replicates of the endonuclease assay of DSIF dependence was ommitted because there was no activity at all, assuming a human error in the preparation of the FluPol promoter mixture.
Replication	Cell-based experiments using luminescence were performed with at least three independent biological replicates, with all attempts at replication being successful. Endonuclease cleavage assays were performed 5-7 times.<br>Protein expression analyses by western-blot, analytical gel filtration, cryo-EM experiments, and elongation assays were performed once.
Randomization	Division of particles into random halves is automatically performed during 3D reconstruction by Relion 3.1.0. Other experiments did not involve randomization.
Blinding	Blinding is not applicable for this study, as group allocation is not used.

Reporting for specific materials, systems and methods

We require information from authors about some types of materials, experimental systems and methods used in many studies. Here, indicate whether each material, system or method listed is relevant to your study. If you are not sure if a list item applies to your research, read the appropriate section before selecting a response.

Materials & experimental systems

n/a	Involved in the study
☐	☒ Antibodies
☐	☒ Eukaryotic cell lines
☒	☐ Palaeontology and archaeology
☒	☐ Animals and other organisms
☒	☐ Clinical data
☒	☐ Dual use research of concern
☒	☐ Plants

Methods

n/a	Involved in the study
☒	☐ ChIP-seq
☒	☐ Flow cytometry
☒	☐ MRI-based neuroimaging

Antibodies

Antibodies used

Primary antibodies for analytical size exclusion:
rabbit anti-Strep-Tag II antibody (ab76949, Abcam)
rabbit anti-RPB3 polyclonal antibody (A303-771A, Bethyl)
Secondary for analytical size exclusion:
anti-rabbit antibody coupled to HRP (NA937, GE Healthcare)

Primary Ab for steady-state levels in cell-based assay:
Influenza A virus PA protein antibody (GTX125932, GeneTex)
Influenza A virus PB2 protein antibody (GTX125925, GeneTex)
Monoclonal Anti-α-Tubulin antibody produced in mouse (B-5-1-2, Sigma Aldrich T5168, lot number 0000089499, 1:10000)
Secondary Ab for steady-state levels in cell-based assay:
Anti-Mouse IgG (whole molecule)–Peroxidase antibody produced in rabbit (A9044, Sigma-Aldrich)
Anti-Rabbit IgG (whole molecule)–Peroxidase antibody produced in goat (A9169, Sigma-Aldrich)

Validation

The antibodies used in study has been evaluated in peer- reviewed publications. anti-RPB3 Ab (Dubbury et al., 2018), anti-PB2 Ab (Krischuns et al., 2024), anti-PA (Da Costa et al., 2015), anti-tubulin (Solinger et al., 2008)
anti-Tubulin
Validation data shown on the provider's website: upon western blot analysis, lysates from control tubulin-negative control worms do not show the expected band, while it is observed for tubulin-expressing worms (Figure 8E, Solinger et al. PLOS Genetics 2008, DOI: 10.1371/journal.pgen.1000820)
anti-PA
A rabbit serum directed against the PA domain (residues 197 to 257) was used to reveal PA expression.
Validation data included in the manuscript: upon western blot analysis, lysates from control mock-transfected cells do not show the expected band migrating at 100 kDa, while it is observed for cells transfected with the PA plasmid.
anto-PB2
Validation data included in the manuscript: upon western blot analysis, lysates from control mock-transfected cells do not show the expected band migrating at 100 kDa, while it is observed for cells transfected with the PB2 plasmid.
anti-Strep-Tag II
Validation data included in the manuscript: upon western blot analysis, a band migrating at 80 kDa in FluPol (Strep-II-tagged) containing lanes. Antibody was validated by the manufacturer by Western Blot analysis used a recombinatly expressed strep-tagged protein in a lysate. A band was only observed if the strep-tagged protein was expressed.
anti-RPB3
Validation data included in the manuscript: upon western blot analysis, a band migrating at 30 kDa in Pol II containing lanes. The antibody was tested by the manufacturer using Western blot analysis and IP elutions against RPB3 using different antibodies as to pull-down RPB3. A band was observed at the expected high if antibodies against RPB3 were used during the IP, but not if a negative control antibody was used.

Eukaryotic cell lines

Policy information about cell lines and Sex and Gender in Research

Cell line source(s)

Hi5 cells: Expression Systems, item 94-002F
Sf9 cells: ThermoFisher, Catalogue Number 12659017
Sf21 cells: Expression Systems, Item 94-003F
The 293T cells were purchased at ATCC (CRL-3216)
The MDCK cells were provided by the National Influenza Center, Paris, France.

Authentication

293T cells were authentified by ATCC using STR profiling. Sex = female. Insect cell were not authenticated in-house. Sf9 cells were tested by the manufacturer by isozyme and karyotype analysis. MDCK cells were not authentified in-house.

Mycoplasma contamination

Not tested for insect cells. The human cell line used has been tested on a regular basis for the absence of mycoplasma, using a specific PCR detection protocol. The human cell line tested negative for mycoplasma contamination

Commonly misidentified lines
(See ICLAC register)

No commonly misidentified cell lines were used in the study.

Plants

Seed stocks	*Report on the source of all seed stocks or other plant material used. If applicable, state the seed stock centre and catalogue number. If plant specimens were collected from the field, describe the collection location, date and sampling procedures.*
Novel plant genotypes	*Describe the methods by which all novel plant genotypes were produced. This includes those generated by transgenic approaches, gene editing, chemical/radiation-based mutagenesis and hybridization. For transgenic lines, describe the transformation method, the number of independent lines analyzed and the generation upon which experiments were performed. For gene-edited lines, describe the editor used, the endogenous sequence targeted for editing, the targeting guide RNA sequence (if applicable) and how the editor was applied.*
Authentication	*Describe any authentication procedures for each seed stock used or novel genotype generated. Describe any experiments used to assess the effect of a mutation and, where applicable, how potential secondary effects (e.g. second site T-DNA insertions, mosiacism, off-target gene editing) were examined.*
